# Image-Based Sensor for Liquid Level Monitoring during Bottling with Application to Craft and Home-Brewing

**DOI:** 10.3390/s23167126

**Published:** 2023-08-11

**Authors:** Josip Musić, Ivo Stančić, Barbara Džaja, Vesna Pekić

**Affiliations:** 1 Faculty of Electrical Engineering, Mechanical Engineering and Naval Architecture, University of Split, R. Boškovića 32, 21000 Split, Croatia; istancic@fesb.hr (I.S.); vpekic@fesb.hr (V.P.); 2 Department of Professional Studies, University of Split, Kopilica 5, 21000 Split, Croatia; barbara.dzaja@oss.unist.hr

**Keywords:** bottling, home-brewing, liquid level measurement, machine vision, optical refraction, optoelectronic sensors

## Abstract

Although craft and home brewing have fueled the beer renaissance in the last decade, affordable, reliable, and simple sensing equipment for such breweries is limited. Thus, this manuscript is motivated by the improvement of the bottle-filling process in such settings with the objective of developing a liquid level sensor based on a novel application of the known optical phenomena of light refraction. Based on the different refraction indices of liquid and air (and critical angle based on Snell’s law), along with a novel LED light source positioning, a reliable liquid level sensor system was built with the aid of an embedded microcontroller. The used operating principle is general and can be used in applications other than the proposed one. The proposed method was extensively tested in a laboratory and limited production settings with a speed of 7 Hz using different liquids and container shapes. It was compared for accuracy to other sensing principles such as ultrasound, infrared, and time-of-flight. It demonstrated comparable or better performance with a height error ranging between −0.1534 mm in static conditions and 1.608 mm for realistic dynamic conditions and good repeatability on the production line with a 4.3 mm standard deviation of the mean.

## 1. Introduction and Motivation

In recent decades, craft brewing has undergone a renaissance [1]: from being an outlier in the beer industry to becoming a dominant niche with 5234 out of 5302 breweries in the USA identifying as craft breweries. In Europe, the beer market has experienced the change caused by the so-called craft beer revolution, especially in regions with a long history of brewing [2,3]. There are thought to be more than 10 thousand craft breweries around the globe [4]. According to the Brewers Association, the craft market share grew from 5.7% in 2011 to 13.1% in 2021 with craft brewers providing more than 172,643 direct jobs [1]. The sector grew double the “standard” beer market rate between 2008 and 2013, with an average of 2.8% [5].

The rise of craft beer is thought to have been fueled by several factors, such as the introduction of “By Local” movements as well as the willingness of consumers to pay more for unique beer taste [6,7]. Its effects are so profound that even some lawmakers [7] have agreed to change their beer laws to facilitate local and regional craft breweries. One of the reasons for the sudden expansion of craft breweries in Europe is that modern consumers choose simple procedures and raw materials that give them a feeling of uniqueness [8]. Recent research for Spanish breweries concluded that the consumption of craft beer does not present health issues for consumers since it is free from mycotoxins, pesticides, pollutant residues, and elemental compositions. In contrast, the same research concluded that 100% of mainstream beers presented at least one mycotoxin, which is another reason to prefer craft breweries [9].

The American Brewers Association (ABA) defines craft beer as one produced by small, independent, and traditional brewery [6], a definition which consumers have interpreted as equal to high-quality products [10]. However, it should be noted that this definition is variable depending on the country. For example, in Italy, craft breweries are considered those not producing more than 170,502 barrels a year, while this threshold is in the USA set to 6 million barrels [6]. Still, in the manuscript, we will refer to craft breweries as those that produce beer on a small(er) scale than industrial ones (regardless of the actual limits). Here, we also include a home-brewing, often considered a seed for craft brewery [7]. Home-brewing is exhibiting an increasing trend in the USA, with around 1.4 million barrels of beer produced by about 1.1 million people in 2017 [11].

One of the main challenges craft breweries faced in their early ages (in the 1980s and 1990s) was the lack of appropriate equipment [6], since much of it was customized for large-scale operation. However, due to the increasing importance of the craft beer market, and the advent of low-cost electronic components, the equipment market for craft brewing is rapidly developing. While some of the developed equipment is available, due to physical dimensions, and other requirements, this equipment might not be suitable for the home-brewing type of producers (or micro-brewers). There have even been some attempts to make the brewing equipment for bottling open source (https://openbook.hr/site/cv.htm (accessed on 18 July 2023)). However, all of the measurement equipment needs to comply with a number of standards and recommendations, including those defined by the *International Organization of Legal Metrology* in [12]. The recommendation extension [13] introduces referent conditions and procedures for testing setups, including those for beer, but within the holding tanks and not inside the bottles during the filling process. Some research on liquid level measuring in tank-type containers is based on such international recommendations [14].

It is also worth noting that the benefits of the Internet of Things have been recognized in the so-called Internet of Beer, which increases efficiency in the processing, delivery, and service areas of beer production [15]. Due to technological advancements, and a decrease in the cost of electronic components, these technologies are becoming accessible to medium and small-sized brewing companies and individuals. Researchers found that off-the-shelf technologies connected to a cloud-connected sensor system, combined with machine learning, could accurately predict alcohol content during fermentation. This is part of the new Industry 4.0 concept, where the digitalization of the manufacturing process leads to an economic benefit for the producer (brewer) by reducing the amount of wasted materials (beer and resources) [16].

While there are several stages of brewing a (craft) beer, from malting to conditioning [17], the manuscript deals with the final stage, which includes bottling and associated sensory systems. It should be noted that, besides bottling, there is also kegging, but this is usually a more complicated process and one that is more expensive and is, in turn, not appropriate for smaller breweries (https://grainfather.com/a-guide-to-bottling/ (accessed on 18 July 2023)). Regardless of the process under consideration, non-contact measurements are preferred due to the hygienic requirements of the brewing and packaging processes (https://cdafrance.com/en/our-packaging-solutions-by-sectors/labelling-machines-and-filling-machines-for-the-beer-industry/ (accessed on 18 July 2023)). Thus, we hypothesize that an image-based system using a special case of optical refraction in conjunction with computationally effective image processing algorithms and of-the-shelf components can lead to an accurate, reliable, and affordable type of sensory system appropriate for liquid level measurement during the bottling process with an application to small-scale breweries.

The remainder of this manuscript is structured as follows. Section 2 gives a brief state-of-the-art review of related fields. Section 3 presents the used experimental setup and the proposed approach’s main operational principles and algorithms. The obtained results are presented in Section 4, where they are also analyzed and discussed. Finally, in the Section 5 a summary of the developed approach is given, along with its observed advantages and disadvantages and some possible ways of improving it.

## 2. Literature Review and Contributions

Today’s food-related industry in all its branches imposes strict process control requirements and regulatory demands. Accurate and reliable level measurements (at all stages of the production process) make for a more straightforward implementation of those demands [18]. These measurements can have different operational principles depending on the type of material being measured (e.g., liquid or a solid), but due to hygienic requirements, should be contactless and should accommodate specifics of the production line (i.e., moving parts like conveyor belts). When considering the beer-filling process, it should be kept in mind that the sensor cannot be attached to the container itself (like it can in tanks). Beer color and bottle size also vary depending on the country and/or producer (https://beerandbrewing.com/dictionary/yIINroTsFH/ (accessed on 18 July 2023)). Finally, additional challenges associated with measuring the liquid level during beer bottle filling are based on the fact that beer foams, thus, are usually filled under CO${}_{2}$ pressurized atmosphere. Also, if bottling is performed after labels have been put on the bottle, it can interfere with the sensor system.

The visual identification of liquid levels is one of the most used application scenarios when dealing with liquids, including a wide variety of applications: from bottle-filling to chemistry experiments [19]. Many examples of machine vision-based approaches in food and pharmaceutical industries can be found in [20,21]. However, there are other measurement techniques available like those based on resistive [22], inductive [23], load or pressure sensor [24,25], radar-based [26,27], capacitive [28], piezoelectric [29], ultrasonic [30,31,32], and fiber-optic transducers [33], as well as ones that are being experimentally tested from a scientific point of view like thermal cameras [34]. Many of these techniques have the disadvantage of being intrusive or having a discrete output (e.g., fiber-optic). Some methods, like the one in [35], use modified techniques to improve performance parameters in particular measurement environments like fuel tanks.

An example of the usage of cameras and machine vision in a large-scale beer production process is, in short, presented in [36], where it is stated that a system named SpeedView from French company R&D Vison has been deployed in the Heineken facility in France. The system uses a Mako G-30 camera with a CMOS image sensor with a global shutter. It is a GigE camera with a maximum pixel resolution of 644 × 484 and a capture speed of 309 fps but using a pixel resolution of 300 × 200 (in a windowing mode). The system processed green Heineken bottles at a rate of 80,000 bottles per hour. The simple use of a vision-based technique in detecting the liquid level in amber glass bottles was presented in [37]. The proposed system applied Python and OpenCV for image processing. The study successfully detected and classified filled bottles into three categories: under-filled, within-target, and over-filled. However, it still needs more development since it cannot address the problem of bubbles.

In [38], a web camera (with 30 fps) and a proximity sensor (for triggering image acquisition) were used alongside an infinite symmetric exponential filter (ISEF) edge detector to detect liquid levels in bottles moving on a conveyor belt. For bottle defect detection, the Hough transform and watershed algorithm in conjunction with the support vector machine are mentioned as possible candidates, while for the edge detection, the same authors in [39] tested three of them (Canny, ISEF, and the Laplacian of Gaussian—LoG) and found that ISEF is the most suitable for bottle liquid level detection. It was noted that different edge detection algorithms could be used depending on whether the bottle defect or liquid level is targeted application.

In [40], the machine vision-based approach for the high-speed monitoring of the quality of olive oil on the conveyor belt and detecting bottle cap anomalies was proposed and tested. Simple image-processing techniques were used: one based on image thresholding and the second one based on edge detection. It is interesting to note that special care was given to light source design and placement for optimal performance. This was performed since darker bottle colors can interfere with liquid edge detection algorithms (as is the case in our research) and additional lighting is needed to accentuate liquid level edges for detection purposes. The used camera had a resolution of 640 × 480 pixels and worked on a 124 fps frame rate, and was placed to capture a side view of the bottle with the LED light source placed on the other side of the bottle. Based on the obtained results, it was concluded that the threshold-based approach was the fastest, but no accuracy results were given (for binary-based classification: Pass/Fail).

Another system based on the optical effects of the measured liquid was presented in [41]. The measurement part of the system consisted of a CMOS camera (with a high frame rate enabling capacity of up to 70.000 bottles per hour) and LED lighting, which could be independently controlled and allowed high-contrast images. When the bottle passed near the proximity sensor at the exact location, it triggered a brief LED flash and image acquisition. Then, based on the transmitted light method, image analysis was performed, and the liquid level was determined. The declared accuracy of the system was up to 99.8% with overfill/underfill from 3 mm (depending on the placement of the system within the production/filling line).

In [42], an offline computer vision-based method was used to track the size and frequency of the occurrence of bubbles in champagne. The proposed approach was based on a photo camera (Olympus OM2 with a 50 mm f/1.8 objectives) and photos that were enlarged ten times. Here, it is of special interest that stroboscopic-type diffused light was used to obtain better-quality images. In [43], a brief overview of computer vision methods for beer quality monitoring is presented. While the paper does not go into methods for the bottling process, it does present basic computer vision techniques (as well as a state-of-the-art review) of the beer quality measures. The authors note that the strongest arguments for using computer vision in evaluating beer’s external quality are its reliability and reproducibility. In [44], similar quality parameters for beer (including foam) were tested and analyzed using modern hardware and data processing algorithms (an ensemble of artificial neural networks).

In [45], a vision-based system (implemented on an ODroid-X2 embedded computer) was proposed for controlling Coca-Cola level during the filling process. The proposed system consisted of two photoelectric sensors for detecting and rejecting the bottle. After bottle passage detection, an RGB image was taken, transformed into HSV format, and analyzed for the fill level. It should be noted, however, that the approach was tested only in a limited laboratory setting and not in the plant itself.

In [46], an integrated system for fill level detection and cap inspection (not limited to the beer/brewing industry) was proposed. While the authors did not present any numeric results, they state that the method could reliably detect the liquid level in a plastic bottle without any label and could detect nine conditions regarding the cap and liquid level. It should be noted that vision-based methods could also be used within (nontransparent) containers/tanks like in [47,48], but these are not in the current focus of our research and thus will not be further reviewed.

Medically related applications can also benefit from the application of machine vision algorithms for liquid-level monitoring. In [49], a system for medical purposes has been developed based on two lasers with different wavelengths for the detection and analysis of liquid meniscus within the container. In such applications, the proper calibration of used glassware is essential, as well as the identification of primary sources of uncertainty [50]. Another example of liquid level monitoring using camera and Sobel filter-based segmentation techniques alongside ellipsoid fitting (for container-size estimation) was introduced in [51]. Here, the camera was placed on the rim of the drinking cup with the aim of monitoring/estimating the patient water intake during the day. The processing took 438 ms per image (160 × 120 pixel grayscale image), resulting in a correlation between the estimated results and the ground truth with the variation of 3% from the mean. The standard error of the mean for ellipsoid fitting was between 4.26 and 4.13 mL.

In [52], a measurement system for liquid level detection in an infusion bottle was proposed. The system was based on the Internet Protocol (IP) camera (HIKVision DS-2CD812PF) placed parallel to the infusion bottle. Liquid level was detected utilizing the edge detection algorithm with the problem of additional horizontal edges being detected (e.g., bottle label). This issue was resolved by observing the dynamic nature of edges. The system was tested with changing light conditions and a tilted infusion bottle with 99% accuracy.

Other areas of applications also use machine-vision-based measurements of the liquid level. In [53], the authors introduced a real-time system for detecting the liquid level and color in a laboratory setting during chemical reactions. The proposed system used a (web) camera with 640 × 480 pixel resolution and was positioned in front of the chemical cylinder at a distance of 36 cm. The approach also used the thresholding edge-detection method and required calibration for pixel-to-metric unit conversion. The obtained results demonstrated an average accuracy of 99.103% with a maximum absolute difference of 0.1130 mL. While the obtained accuracy is good, it was obtained under ideal conditions, as noted by the authors, and it might degrade if additional, more realistic effects are considered. In [19], the computer vision-based method for detecting liquid surfaces and liquid levels for chemistry applications (within the transparent container) was used. The same author updated the approach in [54].

In [33], the authors proposed a continuous method for monitoring liquid levels in cylindrical containers (from 100 to 500 mL). An internally black-coated cylinder container was used, which provides a light wave-guiding structure. The downside was that the coating might dissolve in liquid media and contaminate it. A red light-emitting diode lit the liquid from above, which was detected at the flattened and transparent bottom of the cylinder by a photodetector. The photodetector’s response changes proportionally to the liquid level in the cylinder. The proposed system achieved a measurement resolution in the range of 3.2–14.1 µm and could measure liquid levels over 100 mm in depth. The same authors in [55] proposed a non-contact method for the measurement of the liquid level at a micrometer (40–50 µm) resolution for the liquid volume of up to 47 mL, which is much smaller than in our experiments. The proposed theoretical background of the approach is, however, the closest one to our method: using optically related phenomena due to the different optic proprieties of different media (i.e., air and liquid). Here, depending on the height of the liquid in the 46 mm diameter container, light (emitted from 0.25 mW laser source) covers different distances resulting in circular shapes of different sizes (detected by photodetectors positioned underneath the container) proportional to the liquid level. The authors called this effect the lateral displacement effect. Experimental measurements were performed with a 100 mL container and a maximum of 47 mL effective measurement range using six different mixtures of water and ethylene glycol. Compared to our approach, additional hardware for optical manipulation (splitters and mirrors) was needed with the additional limitation that light needs to be emitted from above the container, which might be impractical or impossible in the bottling process and for closed containers. Additionally, as noted by the authors, the method in [55] is best suited for static and not dynamic conditions and related phenomena (like bubbling) which occur during the filling process.

In [56], the vision-based method was developed for detecting the water content of crude oil in the transparent glassware after centrifuge. The method first transformed the captured image in the YUV format of QVGA resolution. It used the *Y* channel to obtain a grayscale image and used the image grayscale illumination value difference (IGAVD) method to detect actual liquid content. The proposed approach used the OmniVision OV7670 image sensor and STM32F103 32-bit microcontroller, obtaining a measurement error of less than 1%.

It should be noted that cameras and image-based methods are not only limited to indoor settings and cylinder-shaped containers [57,58].

Based on the presented state-of-the-art review and our motivation and hypothesis, the main contributions of the manuscript can be summarized as follows:

- Development of an image-based sensor for monitoring liquid levels in transparent (colored) containers during the filling process, utilizing (a special case of) optical refraction phenomena, made possible by a novel light source positioning (underneath the container). Although the underlying physical principle is simple and known from optics, it is functionally powerful and, to the best of our knowledge, not present in the proposed configuration in the available literature or commercial systems (where the light source is usually positioned opposite the optical sensor, i.e., camera);
- The developed sensor system does not depend on the container shape, color, size, or liquid type, as long as they are both transparent. The proposed sensor system enables insights into the state of the filling process and liquid (which similar methods do not provide) by allowing the possibility of analyzing different optical refraction patterns corresponding to the characteristic cases providing answers to important questions that cannot be easily answered with other sensor systems (e.g., is foaming occurring during the filling and how much, is there bubbling within the liquid during the filling);
- Construction of a self-contained microcontroller-based, working sensor system that relies on the proposed working principles, using low-cost, off-the-shelf components while ensuring a high level of accuracy. This makes it appropriate for small-scale production plants. The system successfully integrated into the open source filling (and labeling) filler;
- Extensive testing of the proposed sensor system operating principle within laboratory and production environments, obtaining results comparable to or better than some other low-cost sensing options while having the possibility of usage even in setups where the container is fully closed (e.g., due to the need for a pressurized atmosphere within the bottle).

## 3. Materials and Methods

Due to sanitary requirements, the sensors for liquid level measurement in the bottle during filling should be non-contact. Also, it should provide the continuous measurement of the liquid level and its numeric values, and not the binary output (i.e., if the level is lower/higher than the predefined threshold). Thus, the additional, general, design requirements were implemented:

- Easily obtainable and affordable components should be used;
- The sensor system should be self-contained with dimensions appropriate for the small-scale production plants;
- Program support should give continuous output values (current state of the level) as well as discrete values if needed (whether it has reached the desired level value);
- The system should be robust to changes in the lighting of the working environment and enable the implementation of several measuring stations working in parallel (without mutual interference);
- The system should be easy to use (especially the alignment phase, if necessary).

### 3.1. Physical Operational Principle

Since image-based techniques were selected to best fit the listed requirements, different optical phenomena were examined to detect the most suitable one. However, other approaches, such as capacitive-based ones [59], were considered and quickly abandoned due to the complexity of realizing continuous measurement and implementation in a production line.

**Figure 1:**
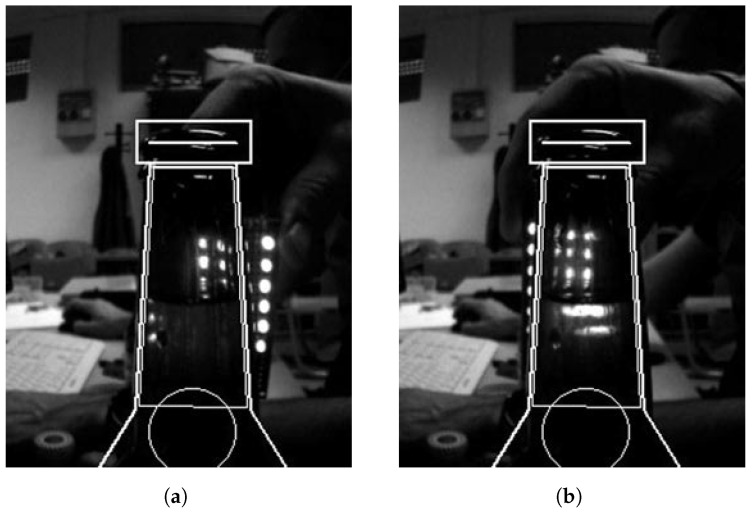
Testing optical phenomena with a 6×3 LED raster light source. (**a**) Beginning of the test. Note that the raster column outside the liquid is visible as a straight line but distorted inside it. (**b**) The liquid level inside the bottle can be estimated based on the location of the distortion within the image of the 6×3 LED raster.

Due to the different refraction indices of liquid and air, image refraction causes a visible optical distortion in the image perceived through the liquid, as depicted in Figure 1.

Please note the bottleneck mask present in the figure, which is not important at this time and will be elaborated on later in Section 3.2.1. The figure shows that a 6×3 LED raster was created in front of which the bottle was positioned or was moving. The size of the raster was selected based on the size of the beer bottleneck but is of no practical measurement importance since the raster is only used to illustrate important optical phenomena.

Due to the refraction of light emitted by the LED raster, the raster image seen by the camera becomes distorted. This distortion is different in areas with liquid, indicating its presence. One can measure the liquid level by detecting the distortion’s location in the image. Depending on the location of the distortion, the effect can manifest itself as a “shift”, as shown in Figure 1a, or blurring, as shown in Figure 1b. Please also note that the detection of a liquid level without the (background) light source is not a trivial task due to the similar bottle and liquid colors in the image as can be seen from Figure 1a.

Considering these initial findings, we discovered that the best position for an LED light source, which ensured a low possibility of interference with environmental and/or neighboring light sources, was underneath the bottle to be measured. This LED positioning also resulted in a more straightforward installation procedure (in terms of integration within the existing filler station), and it ensured direct contact between the light source and the bottle, increasing the system’s robustness. In this case, the refraction phenomenon also occurs, but we exploited a particular (special) case of it. While this phenomenon is well known in physics, due to its importance for our proposed sensor system, we explain it in detail with an example.

First, we introduce Snell’s law which states that, if $n_{1}$ and $n_{2}$ represent indices of refraction for two bordering (transparent) media, and $\Theta_{1}$ and $\Theta_{2}$ are the angles of incidence and refraction that the light ray makes with the normal on the boundary line (between two media) then it holds

(1) $$\frac{\sin\Theta_{1}}{\sin\Theta_{2}}=\frac{n_{2}}{n_{1}}$$

The index of refraction for a particular medium is a dimensionless parameter defined as a ratio between the speed of light in a vacuum and the speed of light in that particular medium. For example, the index of refraction of air is 1.0003, whilst those of water and glass are 1.33 and about 1.5, respectively.

The ray of light that goes into the other media is called transmitted light. It should be noted that, generally, not all light goes from one media to another (refracting in the process), but part of it also reflects back in the same media (at the same angle as the incidence angle). This is referred to as reflected light. Suppose the incidence angle is greater than the so-called *critical angle* ($\Theta_{1_{critical}}$). In that case, the complete ray of light reflects inside the media—a phenomenon known as *total internal reflection*. However, an interesting phenomenon occurs when the incidence angle equals the critical angle in cases when the light passes into the medium with a lower refraction index (i.e., from liquid to air): according to (1), the light bends at 90${}^{\circ }$ angle, skimming the border plane. This light ray then exits the second media at the border level. The critical angle can be derived from (1) and is defined as

(2) $$\Theta_{1_{critical}}=arcsin\frac{n_{2}}{n_{1}}$$

**Figure 2:**
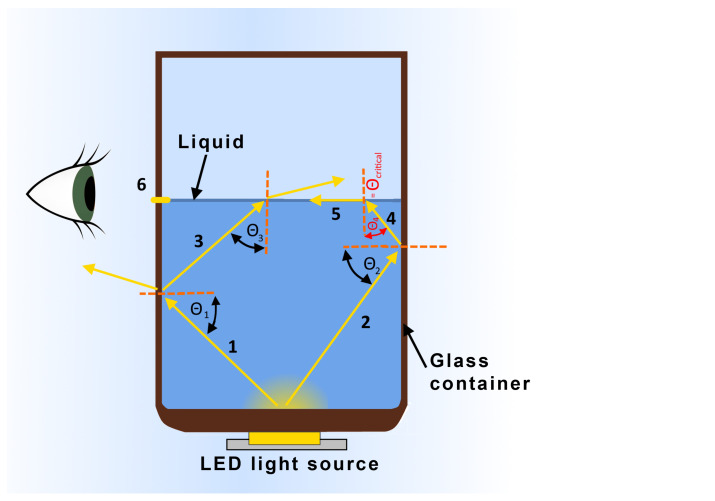
Illustration of light ray reflection and refraction phenomena used for the proposed machine vision system.

With our intended application in mind, a simplified case is illustrated in Figure 2.

In the figure, a LED-diffused light source is located at the bottom of a glass container (for illustration purposes, the thickness of the glass walls is slightly exaggerated). Among many, two light rays are highlighted. One light ray (marked with ***1*** in the figure) goes to the left and hits the liquid-glass surface: part of the ray reflects back inside the liquid (at the same angle; ***3*** in the figure), and part of it is transmitted into the glass. Light ray ***3*** then continues its path through the liquid, hitting the liquid–air surface, and since the incidence angle is smaller than the critical one, part of the ray transmits into the air (with respect to Snell’s law). The other part reflects back into the liquid (for clarity reasons not shown in the figure). Light ray ***2*** in the figure goes to the right, hitting the liquid–glass barrier with the same effect as ray ***1***. However, now the reflected ray (***4***) hits the liquid–air surface at the critical angle, resulting in it entirely being transmitted along the liquid–air border (***5***) back to the glass surface. Since now the incidence angle is 90${}^{\circ }$, the light ray passes through the glass medium in the same path, resulting in a light (***6***) seen by an observer/camera outside the glass container at the height of the liquid level. This can be then used as an easily detectable liquid level marker in the camera image. Please note that during the filling process (or in any real-life situation where level measurement is needed), the surface of the liquid is not ideally still as it is in Figure 2. However, because there are many reflections and transmissions at various angles and not only a critical angle, neighboring angle values also result in light rays going in a similar direction.

**Figure 3:**
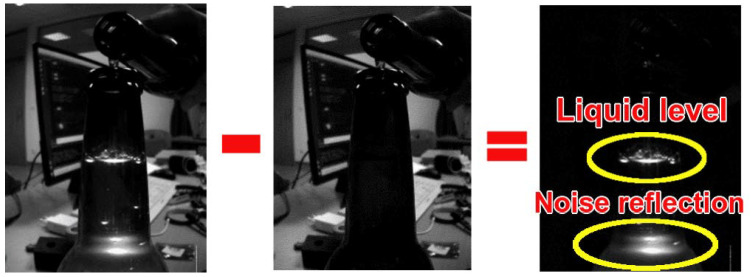
Image differentiation example used in the proposed sensor (PS). Left—image acquired when LED in ON. Middle—image is acquired at the next time instance when the LED is turned OFF. Right—the difference between the left and middle image in which the liquid level is visible, as well as additional noise artifacts.

A real-life example of phenomena illustrated in Figure 2 is depicted in Figure 3. Please note several facts about the figure:

1. There are some additional transmissive effects in the figure due to the bottle shape, which will be addressed later in the manuscript in Section 3.2.1;
2. Liquid (water) was poured from another bottle during the recording of this image, which resulted in splashing and bubbles at the liquid surface which can also be seen through transmissive effects;
3. Cluttered background and other light sources in the background (e.g., computer monitor) do not affect the measurement due to the image differentiation approach;
4. In the image where the LED is turned OFF, it is a very challenging task, even for a person, to detect the liquid level within the bottle.

### 3.2. Experimental Setup and Hardware

System testing was performed in two main setups: laboratory-based (extensive testing) and production-based (simple testing). The laboratory-based testing aimed to ascertain the accuracy of the proposed sensor and compare its performance with other known sensing principles and sensors for level/distance measurement. Please note two following facts. Firstly, when selecting sensors for comparison, their physical dimensions were not considered as a way of integrating them within the filler setup. Secondly, no highly sophisticated (i.e., expensive) sensors were used for comparison since the open–source fillers aim to keep the cost low while providing a high level of performance. On the other hand, limited production-based testing was aimed to showcase that the approach can be applied in a real-life setting with comparable results. The entire filler is still under development and production and thus, full-scale testing in a production environment was not possible.

#### 3.2.1. Laboratory-Based Testing

**Figure 4:**
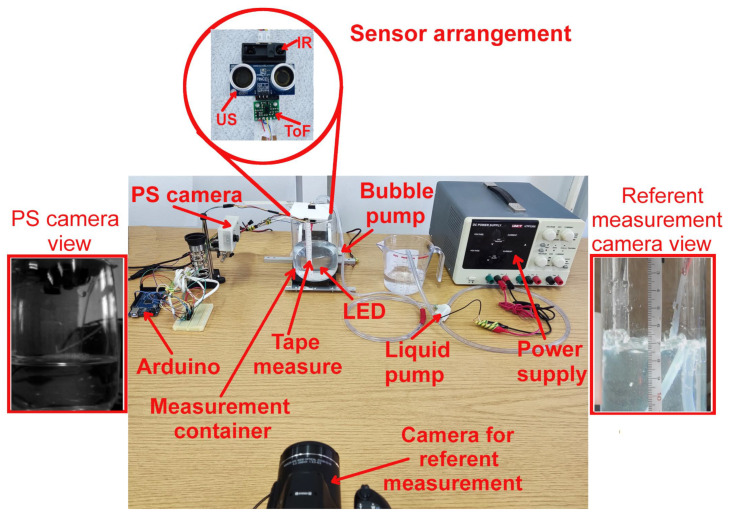
Detailed presentation of used laboratory setup.

The full experimental setup used during the laboratory-based testing can be seen in Figure 4 and will now be described in more detail.

A transparent glass container of 9.53 cm inner diameter was used for testing. The container had vertical walls with a curvature at the bottom. However, the measurement was only performed at a specific part of the vertical wall (cca. 3 cm in height, corresponding to approximately 0.215 L of liquid) due to the easier measurement of the referent value. The liquid used in these tests was water at heights between 6 and 9 cm from the bottom of the container. Please note that the water was used as a test liquid since it does not foam like beer does. The foam would interfere with the measurement of other sensors used for comparison making their results less accurate and reliable. Also, the water refraction index of 1.33 at 20 ${}^{\circ }$C is similar to the one that beer has (between 1.33 and 1.34). This, in turn, preserved the refraction indices relation between glass–liquid–air, making observed phenomena (and thus obtained results) comparable to those of the beer (as is demonstrated later on in the experiment when beer was used).

In order to achieve dynamic measurement conditions, the water was pumped into the measurement container from another auxiliary plastic container (seen in Figure 4 but not annotated) with the help of a generic mini water pump, with a flow rate of 0.1925 L/min (or 3.21 mL/s). As will be explained shortly, a lower filling speed was selected due to timing constraints enforced by other sensors used for comparison. A generic mini aquarium air pump was used to simulate the surface bubble-type phenomena present during actual filling (with a flow rate of more than 2.8 L/min). The container was recorded by a Nikon Coolpix D700 camera positioned at the height of the targeted liquid level in the glass container. The purpose of this camera was to provide a record of the measurement along with LED triggering (which was used to sync all of the measurements) and thus enable a manual offline reference measurement by an experienced practitioner. The camera was set to record the video of the whole experiment in full HD resolution (1920 p) and 30 fps. The recorded video was manually transferred to a personal computer (PC) and analyzed frame per frame in custom-made software that allowed the practitioner to select only frames when LED “flashing” was detected (the moment when the proposed sensor does the measurement) and to manually mark the referent fluid level on the image. Note that a high-power white LED with a power of at least 1 W (10 W recommended) needs to be used for best results. The needed LED power is related to the container’s color, shape, and size.

The liquid level in the glass container was simultaneously measured by four sensors: the proposed sensor (PS) and three sensors mounted on a plastic cover which was placed at the top of the container: PING ultrasonic distance sensor (US), Sharp GP2Y0A41SK0F infrared (IR) sensor, and a VL53LOX time-of-flight (ToF) sensor.

Ultrasonic distance sensors PING (https://www.parallax.com/product/ping-ultrasonic-distance-sensor/ (accessed on 18 July 2023)) is commonly used in mobile robotics for obstacle detection and avoidance but can also be used for liquid-level detection in containers with minimum modifications [60]. Unfortunately, they are impractical in small-diameter containers where their “large” conical-shaped sensing area can misread liquid levels due to the sound reflection from container edges. The size and shape of the container used in our measurement setup were selected with respect to the ultrasonic sensor properties and limitations. Additionally, ultrasonic sensors can be affected by the tilt of a liquid level and ripple, as well as foaming. However, methods for addressing such issues are available in the literature [32]. The measured distance is obtained on the microcontroller by measuring the signal duration and known speed of sound under the current conditions. The useful detection range of the sensor is 2–300 cm with a conically shaped detection area measuring angle of up to 40° (which, according to the datasheet, depends on the distance and target size). The used version requires only a single shared general purpose input–output pin (GPIO) for the echo trigger and signal duration measurement (via a microcontroller-integrated timer), where measurement is executed almost instantaneously after the trigger signal is issued. The recommended timeout during the measurements for the ultrasonic sensor is 60 ms.

Another type of sensor used in our setup was the TOF VL53L0X distance sensor (https://www.st.com/en/imaging-and-photonics-solutions/vl53l0x.html) (accessed on 18 July 2023). The time of flight principle is based on approximating the time it takes a traveling light wave to come in contact with a surface and reflect back to the source. The laser emits the IR (infrared) spectrum, which is invisible to the human eye and offers higher immunity to ambient light. In practice, this sensor can measure absolute distances up to 200 cm, read via digital two-wire interface (TWI). The sensor is set for continuous measurement at 10 Hz with a 33 ms ranging sequence. Generally speaking, ToF sensors can be compared in practice with ultrasonic distance sensors, while they are far superior in tasks involving precise distance measurement with a high refresh rate. The used VL53L0X sensor is not the best-performing ToF sensor in today’s market. Still, its simplicity of implementation, small size, and affordability make them a good choice. Similar ToF sensors have been used in liquid level measurements [61].

The third sensor used in our setup was the infrared beam sensor, namely the Sharp GP2Y0A41SK0F analog distance sensor (https://global.sharp/products/device/lineup/data/pdf/datasheet/gp2y0a41sk_e.pdf (accessed on 18 July 2023)), commonly used as an obstacle detection sensor. However, it was shown to be usable in water level measurements [62]. An IR distance sensor uses a beam of infrared light to reflect off an object to measure its distance, which is then calculated using the triangulation principle with a distance of up to 30 cm. The sensor is analog, which means it outputs a signal in the 0–2.3 V range, which is read using the microcontroller’s integrated AD converter. The relationship between the measured distance and the output analog signal is not linear. Thus, recalculations must be performed on the microcontroller to obtain the exact distance. Additionally, the sensor has relatively large minimal measurement distances (4 cm), which were taken into account during the design of the measurement setup. The aspect that must be considered to obtain a reliable measure is the internal update period of approximately 40 ms, where the sensor may output faulty readings during a short period of recalculation, where we do not have any means of controlling the timing of when an internal update is performed. This issue is handled by executing several readings in a short time and performing median filtering.

Please note that all of these sensors have different housing heights due to their construction and associated printed circuit boards, meaning a constant value offsets their measurements. This value was determined before the measurement (by a caliper) and compensated for during the measurement (i.e., it is not included in the presented results). All the sensors were connected to an Arduino Mega2560 microcontroller board, selected due to its multiple UART interfaces (required for communicating with the PS sensor and the computer) and all other interfaces needed for the sensors. The PS sensor initiates the measurement and sends a trigger signal to an Arduino Mega2560 board, which then schedules the acquisition by other sensors. Note that the MOSFET switch and supporting components (which in turn controlled LED illumination) were integrated on the OpenMV H7 plus cameras customized expansion printed circuit board (PCB).

The PS sensor, through the OpenMV H7 camera (320 × 240 pixels, with integrated ARM Cortex-M7 running at 480 MHz and a fast bus @3.84 GB/s) takes two images in two successive time instances, whereby the LED illuminates the first image. In contrast, the second image is taken under normal conditions (without an additional light source). Then, the differences between these images can be distinguished by simple image subtraction. This effect and its results are shown in Figure 3. In this manner, the PS becomes more robust to light changes in its environment and the environmental light reflections off the container/bottle under measurement.

In our setup, we tested several combinations of M12-compatible lenses with an OpenMV system. The low-distortion lens was an option, that would increase the overall setup price, but instead, we opted for the integrated OpenMV distortions removal option. The camera was initially calibrated using a MATLAB Camera Calibration Toolbox with the aid of an 8 × 7 checkerboard pattern with an 85 × 85 mm cell size. Calibration was performed on the actual image format and resolution (320 × 240 pix) as in the proposed sensor setup to avoid errors due to different image cropping and centering. After calibration, obtained lens distortion coefficients were used to properly remove image distortions in real time, thus increasing the measurement accuracy.

**Figure 5:**
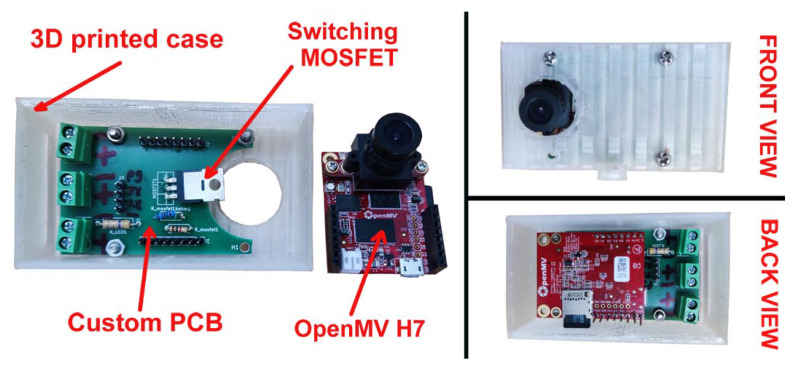
Close-up of the proposed sensor components and their assembly.

**Figure 6:**
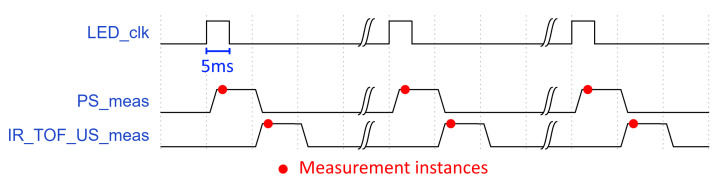
Timing signals during the measurement.

The entire hardware setup of the PS was enclosed in a custom 3D printed case and is depicted in Figure 5. Figure 6 illustrates the complete measurement timing of the sensor. Measurement-enabling signals for US, IR, and ToF sensors were generated 5 ms later to avoid any optical effects on IR and/or the ToF sensor from the LED light source. This time delay, taking into account the water pump in-flow, added a slight error to the US, IR, and ToF measurements, but this had an extremely small value and was thus discarded and not compensated for (i.e., in 5 ms, the pump added 0.016 mL of water, which was an equivalent of 225 μm in liquid height for a given container). Please note that the figure only shows time instances (marked with red dots) for PS at which the liquid level is measured, and not time instances when the second image for image differentiation is obtained (which is not an issue since, in that figure, no liquid level can be detected, as can be seen from Figure 3). One complete processing cycle takes about 150 ms, which should be enough to manage the filling of the whole bottle, which takes approximately 10–30 s. Considering the filling time (30 s) and the target volume of the bottle (500 mL), the average filling speed results in about 16.67 mL/s. With the recording speed of 7 Hz (i.e., seven pairs of images, with and without the LED turned ON), a division shows that the theoretical maximum resolution of the system (not taking into account other effects like the ones associated with the camera) for the particular configuration and filling setup is 2.38 mL.

In the second part of the laboratory-based measurement, only the PS system was used, but this time with a real beer bottle (without any labels) and using a light beer as a liquid. The rest of the setup (minus the air bubble pump) remained the same as in Figure 4. Note that the used beer bottle was dark brown, and a tape measure was attached to its wall for reference measurement. Also, the flow rate was slightly increased (to 0.476 L/min or 7.93 mL/s) to better approximate the actual dynamic working conditions. During the measurement, one unplanned effect occurred (as depicted in Figure 3). Namely, due to different bottle shapes, different reflections can mainly be observed at the base of the bottleneck.

**Figure 7:**
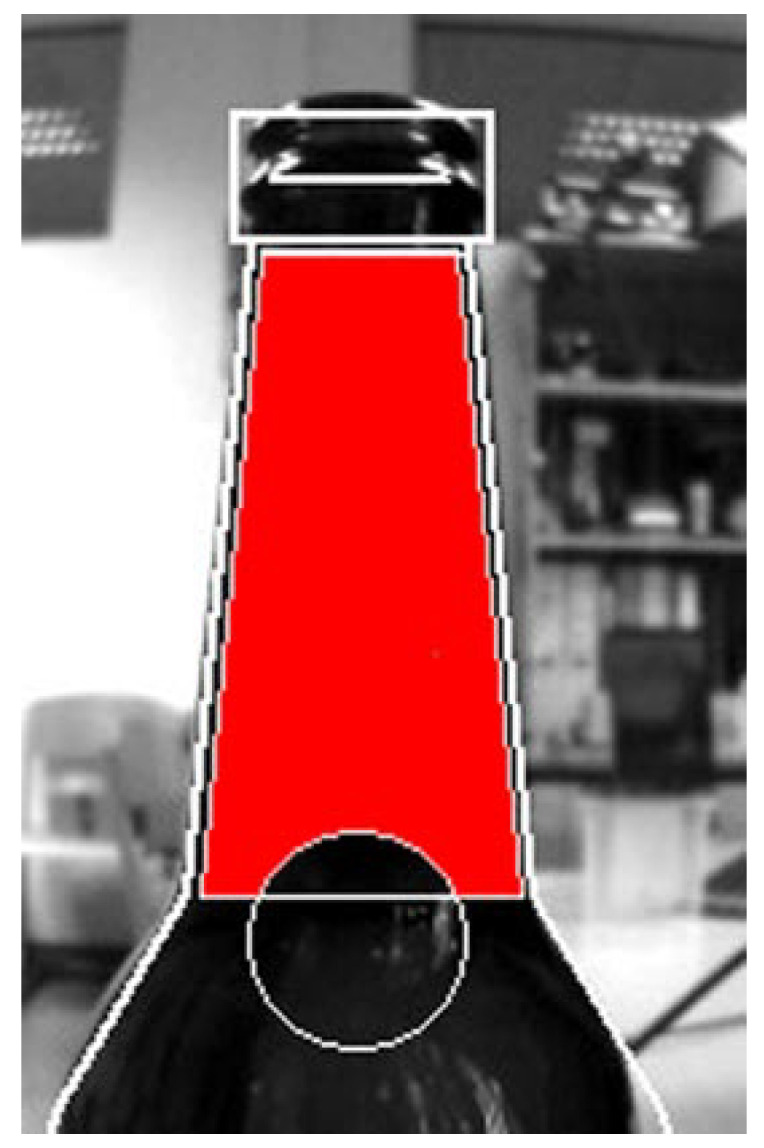
Image mask (in red) defining the measurement area important for completing the bottle filling process.

To eliminate these effects, the (bottom) beginning of the bottleneck is excluded from the analysis, and only the reflections on the rest of the bottleneck are observed with a predefined size, represented by the (red) mask visible in Figure 7. In a standard bottle, this area corresponds to the liquid level between 470 mL and 520 mL. This was possible since the liquid level measurements are most interesting in this part of the bottle since they are used as a stopping condition for the filling process.

**Figure 8:**
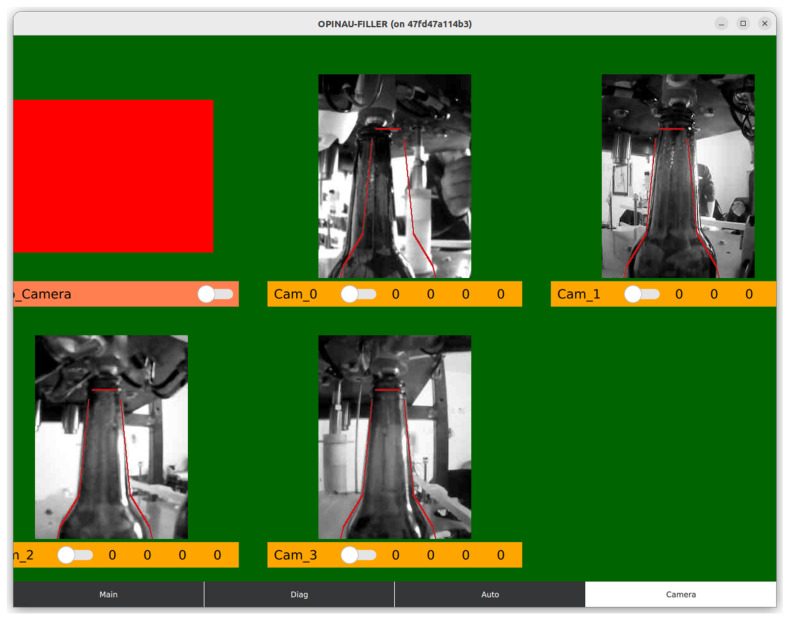
Screenshot of the fill level control dashboard for all five PS cameras used for filling (each camera monitors one filling station). Please note the bottleneck template’s thin red lines in the image, with which the bottle needs to align before the filling process starts. The first camera was offline for this screenshot.

Thus, system–camera alignment is an essential first step when installing the system (performed only once and additionally only if the load-bearing parts of the filling machine move). This alignment implies adjusting the camera and supporting structure so that the bottle (while being filled) is set in the part of the image where the camera expects it to apply masking from Figure 7 accurately. The current control interface (with 5 PS measuring stations) for the filling machine prototype can be seen in Figure 8.

#### 3.2.2. Production-Based Testing

**Figure 9:**
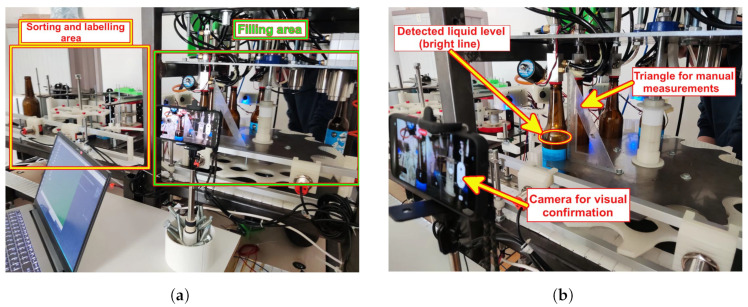
Opinau filler system during the measurements. (**a**) An overview of the complete Opinau filler system with two main parts. For the experiment, we only focused on the filler part. (**b**) A snapshot of measurements during the filling process. Please note that Figure 10 represents an image taken by the camera in this image.

Once the proposed sensor system accuracy was tested in the laboratory-based setting, it was implemented and tested in a limited scope on the filler prototype. The implementation was limited (and mainly focused on repeatability since accuracy was already demonstrated) because the prototype is still under development and is not yet fully operational. An overview of the PS positioning within the filler system and filler area can be seen in Figure 9a. In Figure 9b, a detail from the actual measurements is depicted. Please note that the light transmission line within the bottle (corresponding to the liquid level within the bottle) is visible even from a significantly larger distance than the one at which the PS camera is positioned. The figure also demonstrates that up to five bottles can be simultaneously filled while the bottle is positioned and stationary over the LEDs. This means that the extensive sloshing of the liquid within the bottle during the filling process is not present. Here, a referent measurement camera was a mobile phone camera and a triangle aligned with vertical bottle walls was used for height reference.

**Figure 10:**
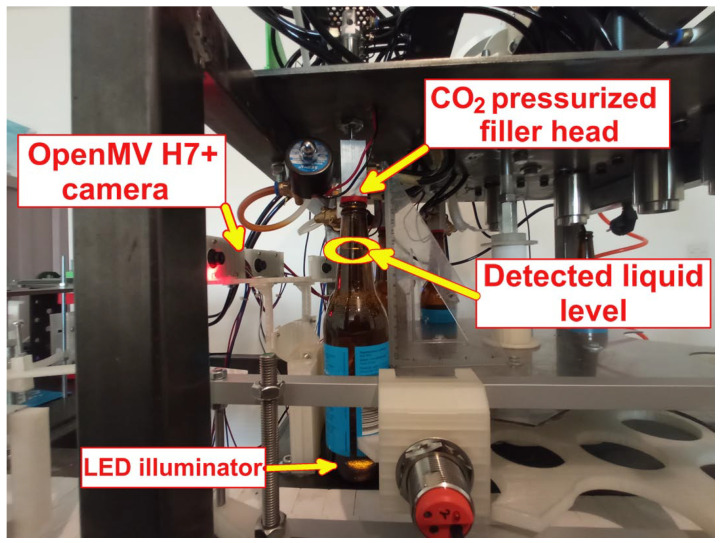
Overview of a single bottle filling in actual production conditions using the proposed system.

An enlarged image of a bottle during the filling process (which is performed with a pressurized CO${}_{2}$ filler head to reduce beer bubbling) is shown in Figure 10. Here, a light transmission line within the bottle can also be seen, as well as the LED source at the bottom of the bottle (ProLight Opto white neutral LED, 720 mA, $P_{max}=27.58$ W). The measurement was performed ten times, with a (constant) set targeted liquid level within the bottle. Note that, due to delays present within the other components of the filling system (outside the proposed sensor, e.g., valve actuator), the error between the actual and desired level was not considered, but the repeatability of the level (under the hypothesis that other components’ delays are quasi constant).

### 3.3. Computer Vision Processing Method

**Figure 11:**
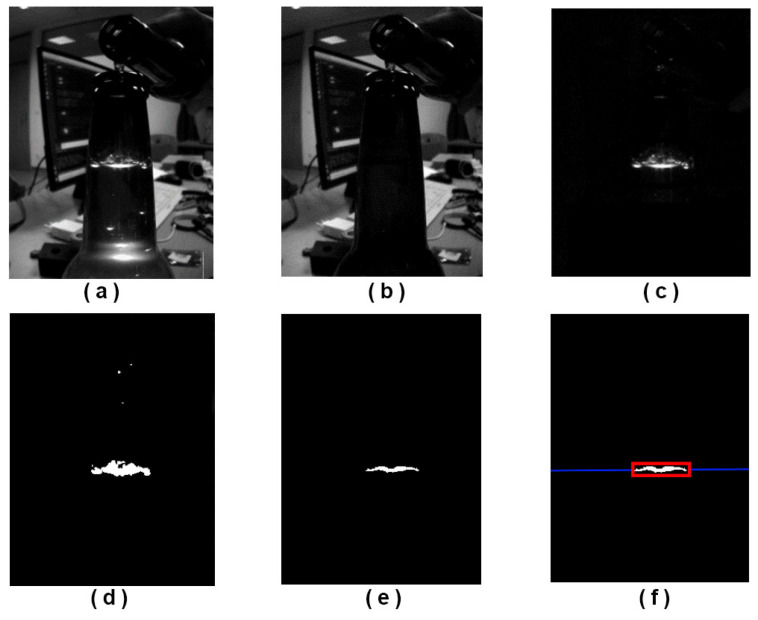
Images used in sub-steps of our proposed fluid level detection algorithm, RAW images (**a**,**b**), processed images (**c**–**e**), and final blob detection result (red rectangle) with fluid fill level depicted with the blue line (**f**).

The proposed method is based on a simple computer vision principle, where the system takes two images, one image where the container is lit with a strong but short flash (as shown in Figure 11a) and one image without extra light (as shown in Figure 11b). The result of the frame differentiation of those two images (along with the application of the bottleneck mask) is another image (Figure 11c) where a horizontal line can be observed due to light diffraction, with few possible extra artifacts due to foaming, bubbles, reflection on bottle base, and similar. After image binarization (Figure 11d), the image is processed with a few (adaptive) morphological operations (to fill discontinuities, remove small blobs, and similar) as shown in Figure 11e. The algorithm searches for all blobs present in images after image processing and finds one which best suits the horizontal line (analyses horizontal vs. vertical ratio). The vertical pixel position which localizes the best candidate blob is, in fact, the position of the fluid fill level in the image, as shown in Figure 11f.

Please note that, before actual measurement, to function properly, the whole system must be calibrated to calculate the exact pixel-to-mm ratio, where the exact mm level is taken from the pre-calculated lookup table. Nonlinear mapping, which creates a lookup table, is calculated from at least ten referent measurements using nonlinear interpolation. The lookup table is unchanged if the setup is also unchanged (camera and container position), which is true when the camera and container filler are fixed in an assembly line. The whole algorithm has to be kept simple and light because it has to be run in real time on a camera-enabled microcontroller with extremely limited resources. Our sensor prototype outputs measured information using an integrated USART interface towards the central computer, which controls another electronic part of the system (all running within the robot operating system framework). The actual output (message) is more advanced and not central to the operation of the proposed sensor system. An example of the provided message is as follows:

<LevelType,HPos,BlobSizeH,BlobSizeW>

where LevelType is the line type described and visualized in Section 4, HPos is line position, BlobSizeH is blob height, and BlobSizeW is blob width.

## 4. Results and Discussion

**Figure 12:**
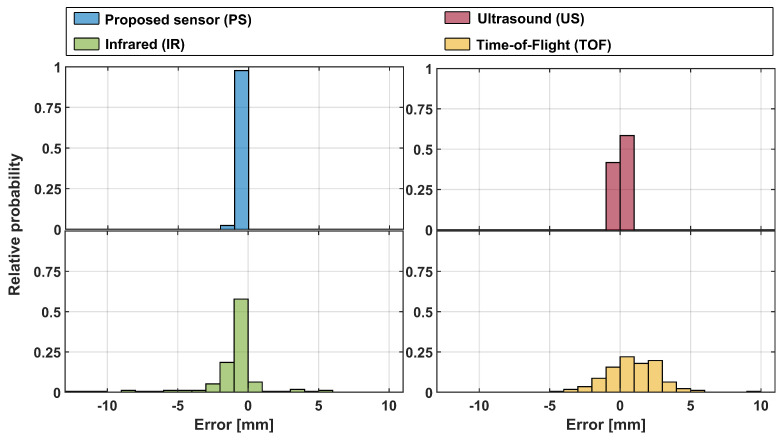
Error histogram of the static liquid level measurements in three different heights within the proposed sensor’s camera field of view with a fixed bin width (1 mm). For comparison, three standard distance measurement sensors are added. Please note that both the *x* and *y* axes in all figures are on the same scale and that *y* is normalized with the total number of bin occurrences.

First, during the laboratory-based measurements, static measurements were performed. This meant that the liquid level in the glass container was kept constant at three different height levels within the camera field of view (FoV) of the container: at the bottom, center, and top of the FoV. This enabled us to establish each sensor’s baseline performance for future comparison. From these measurements, error distribution (with respect to referent camera measurements) was obtained and is plotted in Figure 12. Note that each subfigure has a variable number of bins in the figure, but each bin is of fixed width (1 mm) across all cases (for easier comparison).

The following simple statistic values were obtained for static measurements (173 measurement points for each sensor, i.e., 692 total measurement points). The PS and the US had similar values of simple statistics, with the proposed approach having a slightly tighter histogram better centered around zero error (mean: −0.1534 mm vs. 0.2006 mm, median: 0 mm vs. 0.5 mm, and standard deviation: 0.3497 mm vs. 0.5766 mm). For comparison, two optical sensors (IR and ToF) had somewhat higher statistic values in all categories but were comparable to each other (IR vs. ToF; mean: −0.8570 mm vs. 0.4393 mm, median: −0.2477 mm vs. 0 mm, and standard deviation: 2.3150 mm vs. 1.9598 mm). However, under static conditions, ToF sensors seemed a better choice.

We also performed additional nonparametric statistical testing (Kruskal–Wallis test), which showed that there is a statistically significant effect between observed sensor errors (p<0.0001). Post hoc testing showed a significant difference between all group pairs but two: PS and US (p=0.12) and US and ToF (p=0.87). We believe this confirms conclusions that can be inferred from obtained error histograms: the PS system is the best one but statistically comparable to the US. IR is the worst-performing one, while ToF is statistically comparable to the US. Considering the simple statistical parameters presented above, this is a small surprise but might be due to similar shaped and centered distributions.

**Figure 13:**
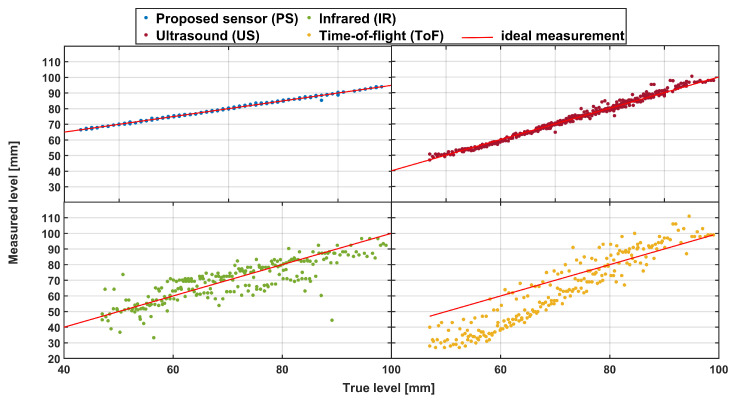
Scatterplot of actual and measured liquid level values (in comparison to the ideal case—red line) obtained with different sensor modules under dynamic, no-noise conditions.

Next, the glass container was filled several times with a steady liquid stream within the targeted measurement height. The summarized results of these measurements can be seen for each sensor type in a scatterplot in Figure 13. The straight red line in the figure represents the ideal case, i.e., when the measurement liquid level value is equal to the actual liquid level.

**Figure 14:**
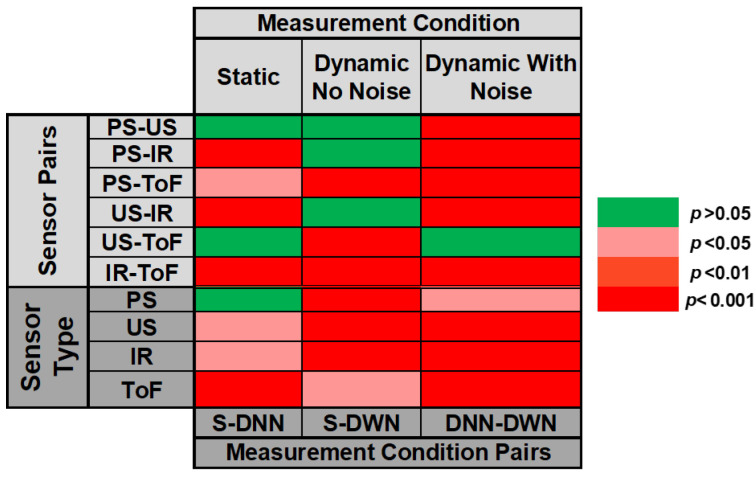
Simplified overview of statistical testing via Kruskal–Wallis for all measurement conditions and sensor types (note that the Kruskal–Wallis null hypothesis at the p=0.05 level tests which whether data come from the same distribution). ST—static condition, DNN—dynamic condition without the noise, DWN—dynamic condition with noise.

The figure shows that the PS is very close to the ideal case and that the US has a similar performance (although with a somewhat broader point spread). The ToF sensor seems to have the worst characteristic and exhibits significant drift when used to measure the fluid level without any modifications. This contrasts with static conditions where the IR sensor performed the worst. Regarding the ToF sensor error, and as described in producer application notes [63], a nonlinearity correction algorithm must be used to improve the ranging accuracy and adjust to the fluid type and container characteristics. This is performed by actual on-site measurement and creating a custom nonlinear mapping for the current setup. Simple statistics are in line with these observations (PS: mean = 0.029 mm, STD = 0.443 mm, median = 0 mm; US: mean = −0.162 mm, STD = 1.553 mm, median = 0 mm; IR: mean = 1.498 mm, STD = 7.496 mm, median = 0.562 mm; ToF: mean = 7.261 mm, STD = 10.935 mm, median = 7.946 mm), as are Kruskal–Wallis tests. A summary of these statistical tests (for this and all other cases considered) is given in a simplified form in Figure 14.

Kruskal–Walis testing demonstrated, on the other hand, that there is only a significant difference between ToF and all other sensors, while there was no significant difference between PS, US, and IR sensors. However, when making this type of comparison, it should be kept in mind that US and IR sensors cannot easily (if anyhow) be integrated into the filler setup since the top of the bottle is not open, and there are no other openings on the beer bottle.

**Figure 15:**
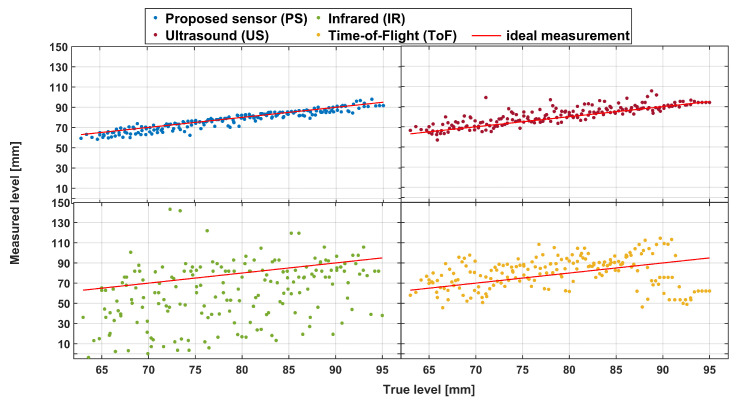
Scatterplot of actual and measured liquid level values (in comparison with the ideal case—red line) obtained with different sensor modules in dynamic, noise-based conditions.

Since some bubbling on the surface of the liquid is expected during the actual filling process, we used an air pump to artificially generate such bubbles. The scatter plots of measurement results using this condition are depicted in Figure 15.

**Figure 16:**
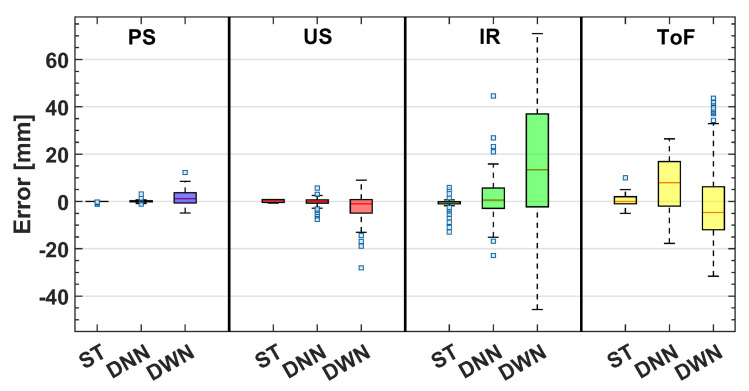
Error box plots for all measurement conditions and sensors tested during the experiments. ST—static condition, DNN—dynamic condition without the noise, DWN—dynamic condition with noise.

As expected, these results constitute degradation compared to those in Figure 13 for all sensors, including the PS. However, this degradation is the least pronounced for PS, as seen from Figure 16. Kruskal–Wallis testing and simple statistics further confirm this. The simple statistic means value increased by 1.579 mm (to 1.608 mm) for PS compared to the increase of 2.194 mm for the US, 16.452 mm for the IR, and a decrease for ToF of 6.101 mm (to −1.160 mm). However, when comparing the medians, PS performs better compared to ToF (1.154 mm vs. −4.638 mm).

These observations are further validated by statistical testing showing that PS (with the best simple statistic parameters) is significantly different (better) than all other tested sensors. This suggests that, while sensor performance is statistically similar for the no-noise scenario, our sensor is the best-performing one under real-life, noisy conditions, demonstrating good robustness in a more realistic case. The only scenario where no statistical difference was observed was the US-ToF comparison.

The final set of testing was performed to examine whether there was any significant difference between individual sensor performances across different pairs of testing conditions (lower part of Figure 14). There was a statistically significant difference (at different *p* levels) for all pairs under testing except for the PS static and dynamic cases with no noise. These demonstrate that our sensor maintains good (and similar) performance under most conditions (which is not the case for other sensors). It is also important to point out the fact that the mean error (in absolute terms) during these measurements resulted in volume error (for the particular container) between 0.164 mL to 11.47 mL (depending on the measurement condition). Considering the targeted measurement volume of 855.97 mL (i.e., 3 cm height), the relative error is between 0.077% and 5.36%.

**Figure 17:**
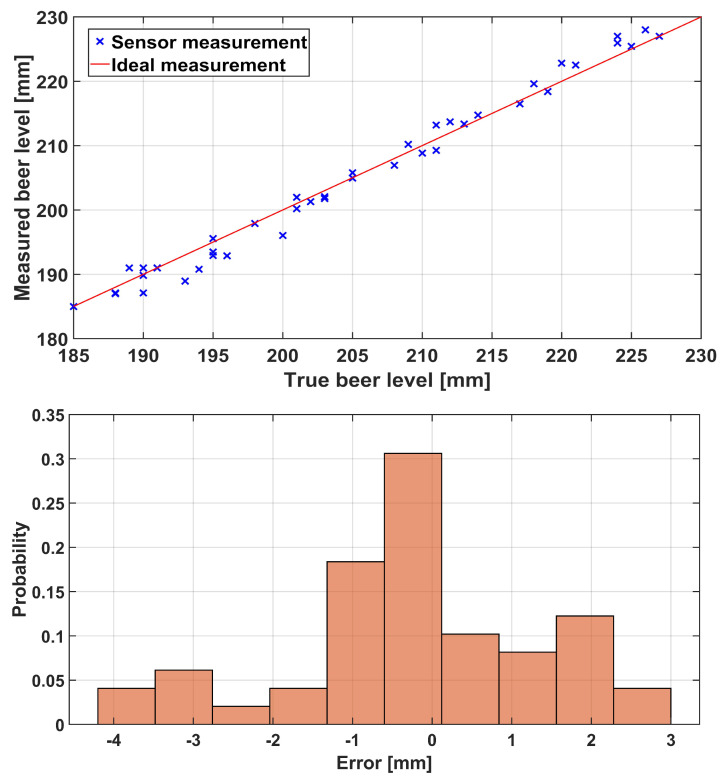
Scatterplot (**upper**) and error histogram (**lower**) for a laboratory measurement of filling beer bottle with beer.

The results presented in Figure 17 were obtained for the PS only during testing with the beer bottle filled with beer as a liquid. This type of measurement included a mask from Figure 7 and was limited to the bottleneck area of the container (as would be in a real-world measurement). From the presented scatterplot, it can be concluded that PS performed comparably with previous results obtained for a different type of container and liquid in it. Please note that these measurements were repeated multiple times since the measurement area (height) was small and the inflow rate was higher, thus resulting in a smaller number of measurement points (even with the increase in the number of LED pulses per second to 5, which was made possible by the fact that there were no other sensors present). A total of 50 measurement points across six measurement runs are thus presented in Figure 17. The error histogram of these measurements is depicted in the lower part of the same figure. The average error was −0.144 mm with a standard deviation of 1.610 mm (and a median of 0 mm). Additionally, we statistically compared this error distribution with error distributions from dynamic conditions (with and without the noise) obtained in previous measurements (presented in Figure 13 and Figure 15) using the Kruskal–Wallis nonparametric statistical test. The test showed no statistically significant difference between the beer and the previous “dynamic no noise” measurements (p=0.7452). At the same time, there was a statistically significant difference between the beer and “no noise” measurement (p<0.005) and the “dynamic noise” measurement (p<0.005). We believe that these results can be interpreted in a two-fold manner: (1.) Since there was some slight foaming occurring during beer-based measurements (i.e., there was some realistic noise present), this might indicate that we injected too much noise during the “dynamic noise” measurement. This is not necessarily bad and illustrates the PS’s robustness even under difficult measurement conditions; (2.) The PS performs consistently for the same and similar measurement conditions regardless of the container shape, measurement range (with the camera’s field of view), and liquid type under consideration. Please note that care was given (since pressurized filler was not used) not to over foam during the measurements. Also, if the mean error is considered, with the mean inner beer bottleneck radius (since the glass wall is not vertical), it translates into an error of about 0.078 mL. If these results (and the results from the glass container and water experiment presented earlier in the section) are compared to some of the results from the literature (absolute error of 0.113 mL from [53], standard error of the mean up to 4.26 mL from [51], underfill/overfill error of 3 mm from [41], and a measurement error of less than 1% from [56]), it can be concluded that they are comparable to them. However, the measurement setups differed in different literature references, which might affect the comparison power.

**Figure 18:**
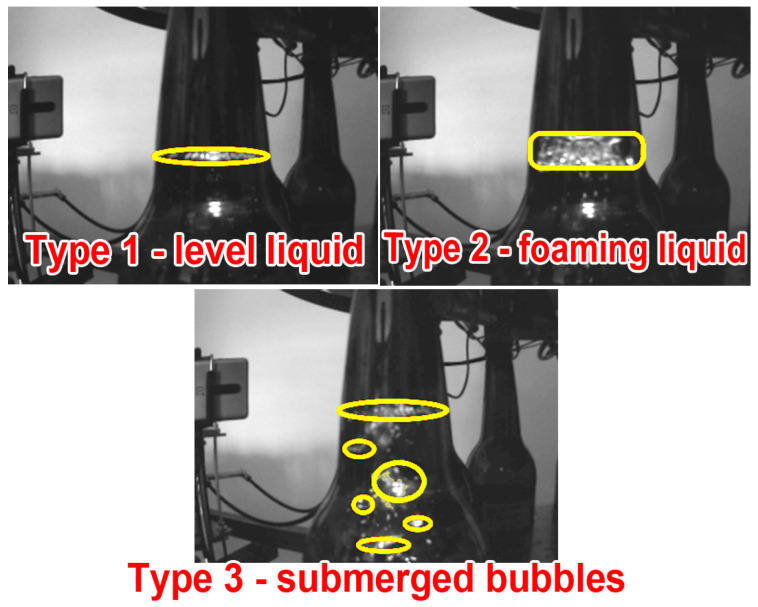
Different types of light patterns were observed during the measurements with a beer bottle.

During system testing, it became clear that the form of reflection the camera can record could be classified into three categories, as shown in Figure 18. Type 1 represents a still liquid level without (or with very little) foam, while type 2 represents a level that has not yet settled since it has a significant proportion of foam. Type 3 characteristic stems from the filling process (air bubbles beneath the liquid surface). This is a novel contribution of our approach, and based on it, machine vision/learning system algorithms could be adapted to detect any of the potential shapes. This is important since, although detecting the level at which the reflection is located (i.e., the liquid level) is precise, it is difficult to accurately measure the beer level in the presence of foam without acknowledging this fact and taking it into account. In our current implementation of the proposed sensor, the foam was dealt with in a way that, when a particular case of it was detected (due to the presence of two separate detection lines), the lower detection line (at the foam bottom) was taken as a level measurement.

**Figure 19:**
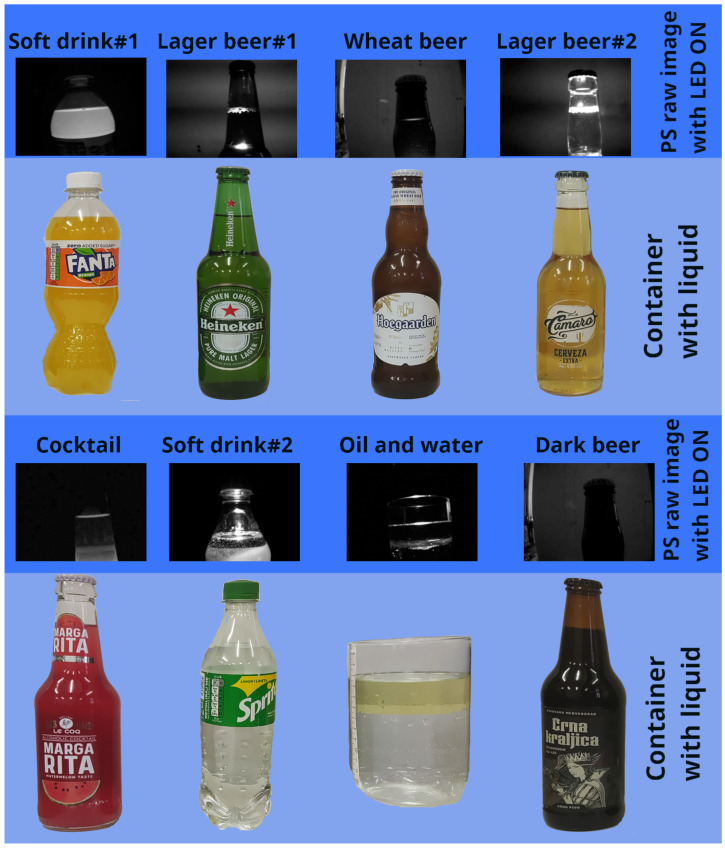
The light pattern required for the successful operation of the PS, observed during the measurement with different bottle shapes and liquids (obtained using OptoSupply OSW4XAHAE1E, a 10 W cool white 850 lm LED source).

It should be noted that different container shapes, container bottom shapes (flat and jagged), and container sizes and materials (glass and plastic) were used for testing to show that the needed optical effect occurs. Testing was performed with different liquids (different colored beers, soft drinks, water, and oil) of different colors and opaque levels. In all but one case (very dark beer), the effect was obtained demonstrating the versatility and applicability of the proposed sensor (but also its limitations). Of special note is the fact that optical effects (i.e., bright lines after image differencing) were obtained even in the case of two different liquids (water and sunflower oil) in the same container, enabling the measurement of individual liquid height. Figure 19 depicts the above-mentioned interesting cases.

**Figure 20:**
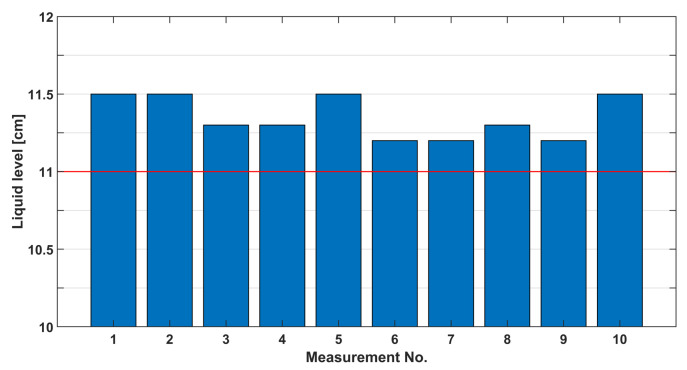
Liquid level measurements in the field. Please note that the red line represents the targeted liquid level.

Finally, the field testing results are shown in Figure 20. The figure shows that the actual level in the bottle was always higher than the targeted one, further affirming the existence of other component delays in the system loop. The amount of “overflow” varies, which can be attributed to several factors, some of which are outside of the proposed sensor domain (e.g., changing CO${}_{2}$ pressure in the container), and some are within it, most dominantly the amount of bubbling and foaming that occurs in the sensor field of view which then requires additional image processing steps to estimate the liquid level which was not part of this research. Regarding repeatability, the standard deviation of all measurements was 0.135 cm (and mean 11.35 cm), and the standard deviation of the mean was 0.043 cm. If it is considered that this variation happens in the lower radius bottleneck, this results in a minor relative volume error, making it acceptable as a filler sensor.

Also, we outline the price of a single sensor (i.e., PS) with the used hardware, but note that this might change since we propose a working principle while using different hardware components (camera, LED, etc.), which might result in different price tags (both higher and lower). In the proposed hardware configuration, the most expensive component is the OpenMV H7 camera (around USD 85 at the time of writing this manuscript), followed by LED at USD 15. With other components (3D printed case, MOSFET transistor, additional resistors, and custom PCB, the PS price tag came at around USD 120. In comparison, commercial/industrial solutions use cameras that have a price of up to USD 2000 (in the case where NVidia Xavier NX is embedded). Similar research prototypes often employ embedded computers like Raspberry Pi with a price tag of about USD 130 (not considering the camera). All of this highlights the low-cost nature of the proposed sensor system.

## 5. Conclusions

The manuscript proposes a novel sensor for measuring the level of a liquid in a transparent glass container, aiming at the bottling process of small-scale home and craft beer which introduces several additional design requirements that the proposed sensor successfully addresses (like sanitary requirements and the fact that pressurized filler is used, blocking the opening of the bottle/container, etc.). While the working principle is straightforward, to the best of our knowledge, a similar design does not exist in the commercial setting or scientific literature. The standard production lines usually employ opposing LED sources (to the camera) for the verification of the fill level but not level measurements during the actual filling/bottling process. This is because the occurrence of multiple optical effects might interfere with the computer vision algorithm performance if the bottle is not positioned precisely and if in the presence of a beer-filling stream. However, please note that in the case of the opaque liquid (i.e., very dark beer), our approach (with the current LED source configuration) will fail, while opposing-side methods will yield a result. Thus, a combination of the two systems might be an interesting approach.

The proposed sensor was extensively tested in laboratory and production settings, with different container shapes, colors, and various liquids (water and beer). Tests also compared the proposed sensor to other sensor types that might be considered in the proposed setting (low-cost, home brewery) and demonstrated a consistently better performance under different conditions (which was, in most cases, statistically significant). The proposed approach demonstrated several advantages, compared to other similar sensors that might be used, but also some drawbacks (which we believe to be fewer in number). The benefits of the proposed sensors include high accuracy, good repeatability, robustness to noise, simple working principle, low price, ease of installation to existing production lines, and ease of maintenance. Additionally, the output results can be used for more detailed and in-depth analysis (like the level of foaming and similar). Drawbacks include the need to precisely position the bottle during the measurement procedure to eliminate residual reflections, the price of the current prototype that could still be slightly high for home brewers, and the requirement for additional power supply and components for installation in the existing filler system. Due to the sensor system design, which measures the liquid level from the front side, and the fact that only the edge of the liquid level is illuminated and visible on camera, the measurement process is not disturbed by the liquid that is poured into the container. Also, the approach could be used for measuring the height of several different liquids (with different densities) in the same container since the thickest liquid goes to the bottom of the container (where the LED light source is), which enables the usage of the special refraction phenomena that the proposed algorithm is based on.

Several possible improvements and future research directions have been identified during the current system version’s development. First, using the same operating principle, a higher frame rate could be achieved to accommodate a higher filler rate for industrial-orientated scale production, which would require more advanced hardware. The inclusion of machine-learning algorithms within the proposed system, and with available input parameters from other plant subsystems, could upgrade the approach to recognize working conditions (i.e., consider the delay of other equipment in the filler process) and predict the stopping of the filling process on time for the targeted liquid level.

## Data Availability

Not applicable.

## Abbreviations

The following abbreviations are used in this manuscript:

ABA	American Brewers Association
AD	Analog-to-Digital
DNN	Dynamic Condition Without the Noise
DWN	Dynamic Condition With Noise
FoV	Field of View
FPS	Frames per Second
GPIO	General Purpose Input–Output Pin
HD	High Definition
HSV	Hue, Saturation, and Value
IGAVD	Image Grayscale Illumination Value Difference
IP	Internet Protocol
IR	Infrared
ISEF	Infinite Symmetric Exponential Filter
LoG	Laplacian of Gaussian
LED	Light-Emitting Diode
MIPI	Mobile Industry Processor Interface
MOSFET	Metal-Oxide-Semiconductor Field-Effect Transistor
MP	Mega Pixel
NoIR	No Infrared
S	Static
STD	Standard Deviation
PC	Personal Computer
PCB	Printed Circuit Board
PS	Proposed Sensor
QVGA	Quarter Video Graphics Array
ToF	Time-of-Flight
TWI	Two-Wire Interface
UART	Universal Asynchronous Receiver/Transmitter
US	Ultrasonic
USA	United States of America
USD	United States Dollar
YUV	Luma, Blue Projection, Red Projection
